# Economic evaluation of diagnostic sleep studies for obstructive sleep apnoea: a systematic review protocol

**DOI:** 10.1186/s13643-021-01651-3

**Published:** 2021-04-09

**Authors:** Andrea N. Natsky, Andrew Vakulin, Ching Li Chai Coetzer, R. D. McEvoy, Robert J. Adams, Billingsley Kaambwa

**Affiliations:** 1 Department of Health Economics, College of Medicine and Public Health, Flinders University, Bedford Park, South Australia Australia; 2 National Centre for Sleep Health Services Research: A NHMRC Centre of Research Excellence, Flinders University, Adelaide, South Australia; 3 Adelaide Institute for Sleep Health, College of Medicine and Public Health, Flinders University, Adelaide, South Australia Australia; 4 Sleep and Circadian Research Group, Woolcock Institute of Medical Research, University of Sydney, Camperdown, New South Wales Australia; 5 Respiratory and Sleep Services, Southern Adelaide Local Health Network, SA Health, Bedford Park, South Australia Australia

**Keywords:** Obstructive sleep apnoea, Cost-effectiveness, Economic evaluation, Sleep breathing disorder, Systematic review

## Abstract

**Background:**

Obstructive sleep apnoea (OSA) is a significant public health problem affecting a large proportion of the population and is associated with adverse health consequences and a substantial economic burden. Despite the existence of effective treatment, undiagnosed OSA remains a challenge. The gold standard diagnostic tool is polysomnography (PSG), yet the test is expensive, labour intensive and time-consuming. Home-based, limited channel sleep study testing (levels 3 and 4) can advance and widen access to diagnostic services. This systematic review aims to summarise available evidence regarding the cost-effectiveness of limited channel tests compared to laboratory and home PSG in diagnosing OSA.

**Methods:**

Eligible studies will be identified using a comprehensive strategy across the following databases from inception onwards: MEDLINE, PsychINFO, SCOPUS, CINAHL, Cochrane Library, Emcare and Web of Science Core Collection and ProQuest databases. The search will include a full economic evaluation (i.e. cost-effectiveness, cost-utility, cost-benefit, cost-consequences and cost-minimisation analysis) that assesses limited channel tests and PSG. Two reviewers will screen, extract data for included studies and critically appraise the articles for bias and quality. Meta-analyses will be conducted if aggregation of outcomes can be performed. Qualitative synthesis using a dominance ranking matrix will be performed for heterogeneous data.

**Discussion:**

This systematic review protocol uses a rigorous, reproducible and transparent methodology and eligibility criteria to provide the current evidence relating to the clinical and economic impact of limited channel and full PSG OSA diagnostic tests. Evidence will be examined using standardised tools specific for economic evaluation studies.

**Trial registration:**

**PROSPERO (CRD42020150130):**

**Supplementary Information:**

The online version contains supplementary material available at 10.1186/s13643-021-01651-3.

## Background

Obstructive sleep apnoea (OSA) is a condition characterised by airflow cessation or obstruction caused by complete or partial collapse of the upper airway, which affects 9 to 38% of the general adult population [1, 2]. Unfortunately, underdiagnosed OSA is common, occurring in 75 to 90% of individuals in the US general adult population and adult surgical patients [3–5]. In Australia, studies have reported up to 10% of individuals remain undiagnosed and untreated in the community [6, 7]. When left untreated, individuals with OSA are at heightened risks of metabolic syndrome [8–11], cardiovascular diseases [12–14], neurocognitive abnormalities [15, 16], mental health problems [17], behavioural alteration [18], traffic collisions [19], workplace accidents and productivity losses [20–22], reduced quality of life [23, 24] and premature death [25–28]. The high prevalence and various adverse health outcomes associated with OSA contribute to significant economic costs [29–31]. In Australia, OSA accounts for $408.5 million of total health system costs and $2.6 billion of indirect financial costs, making it the highest contributor to sleep disorder-related expenses [32].

The health and economic burden related to OSA can be diminished with effective and efficient case-identification and treatment. The latter is readily available and found to be cost-effective [33–35]. A recent systematic review that investigated the impact of OSA therapies suggested that treating OSA has a positive effect on quality-adjusted life-years (QALYs) and can reduce health care utilisation and costs [36]. Despite the existence of effective treatments, case identification of OSA remains a challenge and creates the need to widen diagnostic access for those with suspected OSA [37]. The current gold standard for diagnosing OSA is full overnight laboratory polysomnography (PSG, i.e. level I study)) [38–40]. However, laboratory PSG is expensive, time-consuming and labour intensive, which creates further access barriers to OSA diagnosis [38–40]. Researchers have investigated alternatives to laboratory PSG, which include portable PSG (i.e. level 2 [L2] study) and limited channel testing (i.e. level 3 [L3] and level 4 [L4] studies) that are typically performed in the patient’s home. While full PSG typically (L1 and L2 studies) utilises more than seven recording channels, L3 (polygraphy) measures four to seven channels, while L4 uses only one to three physiological measures to derive a diagnosis [41], making them less labour intensive and less time consuming to report. Previous studies have demonstrated favourable results showing a high level of agreement between limited-channel testing and in-laboratory PSG, particularly when diagnosing moderate to severe OSA [42, 43].

Limited channel tests can advance and widen access to diagnostic services and treatment initiation in OSA, ultimately reducing the substantial economic burden related to OSA. However, to date, research into their cost-effectiveness remains limited and contentious. Scarce evidence exists for the cost-effectiveness of L4 devices despite results suggesting that a single-channel sensor offers a cheaper alternative to polysomnography [44]. Previous economic evaluation models assessing the economic impact of polygraphy (i.e. L3 testing) relative to PSG using published data showed contradictory results [45–47]. However, several empirical studies found consistent results signifying similar effectiveness and lower costs of L3 studies vs. PSG [48–53]. Despite the longstanding controversy of the value and efficiency of polygraphy, the test was approved by the US Centres for Medicare and Medicaid Services and the American Academy of Sleep Medicine practice parameters for use in selective populations in 2008 [54, 55]. Following the change in the health insurance policy that enables reimbursement for home sleep polygraphy in the USA, there has been a growing demand for limited-channel testing for OSA [56]. However, L3 tests are still not compensated by the Medicare Benefits Schedule in Australia where access to OSA diagnosis remains restricted [57].

Robust evidence about the potential economic benefit of OSA diagnostic testing is required to guide decision-makers towards better allocation of scarce healthcare resources. Only a few studies have sought to investigate the health economic impact of OSA diagnostic studies [58, 59]. One previous literature review included nine studies, predominantly examining the cost of diagnostic testing [58]. In contrast, the most recent review has focused on constructing a cost-effectiveness model of OSA management based on existing data [59]. However, none have thoroughly examined the evidence related to the cost and effectiveness of limited channel testing compared to PSG. Hence, we aim to address this gap in the literature by conducting a structured systematic review of economic studies to capture, critically appraise and synthesise data related to health economic outcomes of OSA diagnostic tests, focusing on L3, L4 and PSG testings (LI and L2) in adult OSA populations. Our review will generate information that will help decision-makers determine whether implementing limited channel testing provides value for money for OSA case identification.

## Methods

This protocol has been registered with the PROSPERO database (registration number CRD42020150130) and is being reported in accordance with the reporting guidance provided in the Preferred Reporting items for Systematic Reviews and Meta-Analyses Protocols (PRISMA-P) statement [60] (see checklist in Additional file 1).

### Inclusion criteria

#### Population

This review will include studies of individuals aged 18 years or older. There is no maximum limit of age.

#### Intervention of interest and comparator

The intervention of concern includes limited channel tests (L3 and L4 studies), while the comparator will be attended and unattended PSG (L1 and L2 studies). The limited channel tests utilise lesser channels that are typically part of the PSG. L3 assessment uses at least four channels, including measure of airflow, heart rate, respiratory movement and oximetry [61]. L4 usually employs single or two-channel system, including oximetry [61]. However, for the purpose of this review, any limited channels system that does not meet the minimum benchmark for L3 study will be categorised as L4.

#### Setting

Although limited channel systems are mainly designed to be conducted unattended in the patient’s home, they can also be used for attended studies in the laboratory. As the review is focused on the use of limited channel tests, there will be no restriction as to where the sleep study took place. Studies that compared full PSG and limited-channels tests either in a laboratory or at a patient’s home, unattended or attended will be considered for inclusion.

#### Study design

The systematic review will consider both model and non-model based full economic evaluation studies (i.e. cost-minimisation analysis [CMA], cost-effectiveness analysis [CEA], cost-benefit analysis [CBA], cost-consequence analysis [CCA] and cost-utility analysis [CUA]) that investigate limited channel studies versus polysomnography.

#### Costs and outcome measures

Expenses related to the interventions, resource use, effectiveness and cost-effectiveness of OSA will be reported in the review. In terms of cost-effectiveness measures, the units will vary according to the study design. The findings for CEA and CCA studies will generally be expressed in terms of cost per unit of effect in clinical outcome with those for CUA expressed as QALY and disability-adjusted life years for CUA [62]. The net benefit ratio will be reported for CBA studies [62]. The outcome measurements for the CEA, CCA and CUA can be summarised in the form of an incremental cost-effectiveness ratio, that is generated by dividing the difference in costs by the outcome relative to the study [62]. The probability of the cost-effectiveness of targeted intervention at a different monetary threshold, typically derived from the cost-effectiveness acceptability curve, will be reported in the case where insufficient reporting of studies were found. This review will include all cost perspectives as reported by identified studies including societal, patient and third-party payers (e.g. government, health insurance, employer).

#### Language

The review will include studies published in English.

### Exclusion criteria

Studies that include children, adolescents (< 18 years old) and non-human subjects will be excluded. Exclusion criteria will also extend to theory papers, letters, conference proceedings, theses, dissertations, reviews, editorials and studies where full texts could not be attained.

### Information sources and search strategy

Several steps will be undertaken to find economic evaluation studies on OSA diagnostic testing. First, we will conduct a preliminary search through MEDLINE, focusing on the titles, abstract and index terms to develop key terms for three key pre-defined concepts relating to the research question.

The first concept is related to sleep apnoea and sleep breathing disorders. The second term is related to OSA diagnostic testing, which includes polysomnography, polygraphy, limited channels, home sleep apnoea test, portable monitoring and home sleep test. While economic evaluation, QALY, quality of life, cost analysis, CMA, CCA, CUA, CEA, CBA, healthcare costs and economic model built the third search concept.

Following the identification of keywords and index terms, we will conduct a full search using the developed search strategy connected by the ‘AND’ operator across the following databases from inception onwards: MEDLINE, PsychINFO, Emcare, Cochrane Library, SCOPUS, CINAHL, Web of Science Core Collection and ProQuest databases (see Additional file 2). A minimum of one key concept must be present in the search result. Eligible studies will be selected through screening of the title, abstract and full-text appraisal by at least two reviewers. Cross-reference checking of relevant identified articles will be undertaken as the final step of the search strategy. EndNote X9 (©2020 Clarivate Analytics) and Covidence (©2017 Covidence) will be used to manage data throughout the initial search, quality assessment and data extraction.

### Study selection

At least two independent reviewers will select studies based on the eligibility criteria. If conflict or disagreement arises between the reviewers, a research team discussion will be conducted to resolve issues until a consensus is reached.

### Data extraction and synthesis

The reviewers will extract data from included papers using the Joanna Briggs Institute (JBI) Data Extraction Form for Economic Evaluations [63]. Descriptive data extracted will be comprised of information on the study population and setting, intervention and comparator, economic evaluation methods, analytic perspective(s), source of effectiveness data, prices and currency, analytical duration, cost-effectiveness method, sensitivity analysis, measures of resource use, cost and health outcome(s). Furthermore, study findings on healthcare utilisation effectiveness, cost and cost-effectiveness measures will also be reported. The authors will then discuss factors that promote or reduce the cost-effectiveness of OSA diagnostic testing according to the comparative results of the included economic studies.

Data extracted from relevant papers will first be analysed and summarised qualitatively using the JBI Dominance Ranking Matrix [63], which displays three-by-three matrix with three classifications of economic studies: strong dominance, weak dominance and non-dominance. Subgroups analysis will be undertaken in terms of different sleep study types and comparators (e.g. L1 vs. L3, L2 vs L4), as well as the severity of OSA (e.g. mild, moderate, severe) where relevant.

If the nature of data extraction allows for aggregation of results in terms of quality, quantity and contexts of the identified studies, where possible economic findings (i.e. differences in cost and ICER between intervention and control groups) in relevant subgroups will be synthesised using meta-analysis in Stata 16 [64]. Clinical outcomes that can be expressed as binary outcomes will be reported as odds ratios with a 95% confidence interval (CI), whilst continuous data such as cost and ICER will be reported as mean differences (MD) along with its 95% CI. The OR and MD estimates will be calculated using an appropriate technique (e.g. the Mantel-Haenszel method [65] for categorical binary outcomes and random or fixed-effect models [66] for continuous data). The *I*^2^ statistic will be used to measure heterogeneity, with a value of 85% or higher indicating significant heterogeneity [67]. Financial costs reported in the study will be presented in 2020 Australian Dollars (AUD) with originally published costs in parentheses. Results originally published from non-AUD currencies will be converted to AUD for the publication year using purchasing power parity and then adjusted for inflation using a health-related inflation index where relevant.

### Risk of bias (quality) assessment

Methodological quality and validity checks of the included economic evaluation studies will be conducted by two reviewers primarily using JBI Critical Appraisal Checklist for Economic Evaluation [68], a quality assessment tool based on the guidelines developed by Drummond et al. [62]. An additional checklist by Philips et al. [69] will be utilised specifically for economic studies using a modelling study design or hypothetical cohort. Since economic evaluation studies often employ various cost perspectives and report distinctive health economic measures in different contexts and regions, the European Network of Health Economic Evaluation Databases checklist will be used to further assess generalisability and transferability of included studies [70].

## Discussion

The proposed systematic review will be reported in accordance with the reporting guidance provided in the Preferred Reporting Items for Systematic Reviews and Meta-analyses (PRISMA) statement [71]. This systematic review will present current evidence relating to the economic impact of OSA diagnostic testing methods (i.e. limited channel versus full sleep study testing) using a reproducible and transparent systematic literature search. We have outlined comprehensive inclusion and exclusion criteria of eligible studies, which encompass study design, context, target population, intervention and comparator, cost and outcomes measures. A search strategy and a list of databases for searching have been determined, followed by a strategy for data extraction and synthesis. Included studies will be subjected to critical appraisal using standardised tools tailored for model and non-model based economic evaluation studies. Some potential limitations of this review include the heterogeneity of findings and cost perspective, which can influence the aggregability, transferability and generalisability of extracted data. Another limitation is information bias originating from our restriction to studies reported in English language and adult populations. However, this review is timely in addressing and identifying gaps in current evidence regarding diagnostic options for OSA. Our findings can potentially inform evidence-based decision making when implementing limited channel sleep study testing, particularly in Australia, where undiagnosed OSA and constrained access to diagnostic services remains an important issue.

## Supplementary Information

**Additional file 1.** PRISMA-P 2015 Checklist.

**Additional file 2.** Search strategy draft for main electronic database.

## Data Availability

Not applicable

## Abbreviations

AUD: Australian Dollar
CBA: Cost-benefit analysis
CCA: Cost-consequence analysis
CEA: Cost-effectiveness analysis
CI: Confidence interval
CMA: Cost minimisation analysis
CUA: Cost-utility analysis
JBI: Joanna Briggs Institute
L1: Level one (attended polysomnography)
L2: Level two (unattended polysomnography)
L3: Level three (polygraphy)
L4: Level four (single to three-channel OSA diagnostic test)
MD: Mean difference
OR: Odds ratio
OSA: Obstructive sleep apnoea
PRISMA-P: Preferred Reporting Items for Systematic Reviews and Meta-Analyses statement
PSG: Polysomnography
QALY: Quality-adjusted life years
USA: United States of America
