# Supplementary material for: Economic evaluation of diagnostic sleep studies for obstructive sleep apnoea: a systematic review protocol
Source: Syst Rev. 2021 Apr 9;10:104. doi: 10.1186/s13643-021-01651-3 (PMC8035771; doi:10.1186/s13643-021-01651-3)
Supplement: Supplementary file 2 — Additional file 2. Search strategy draft for main electronic database. [file 13643_2021_1651_MOESM2_ESM.docx]

## Additional file 2

### Search strategy draft for main electronic database

### MEDLINE

| 1 | exp economics/ or exp Decision Trees/ |
| --- | --- |
| 2 | "costs and cost analysis"/ or cost-benefit analysis/ or "cost of illness"/ or health care costs/ |
| 3 | ((economic* adj1 (analysis or evaluat* or model*)) or (cost adj2 (effective** or utilit* or benefit or analysis or minimi*))).mp. or ("quality adjusted life year*" or qaly).tw,kf. |
| 4 | or/1-3 |
| 5 | sleep apnea syndromes/ or exp sleep apnea, obstructive/ or Snoring/ |
| 6 | (apnoea or apnea or hypopnea or hypopnoea or snore or snoring).tw,kw. |
| 7 | ("upper airway resistance" or "sleep disordered breathing").tw,kw. |
| 8 | ((sleep related or sleep-related) adj2 breathing disorder*).tw,kf. |
| 9 | or/5-8 |
| 10 | Polysomnography/ |
| 11 | ((Limited-channel or limited channel or sleep or apnoea or apnea or portable or ambulatory or home) adj3 (study or test or device or diagnos* or monitor* or manage* or identif*)).tw,kf. |
| 12 | (Polysomnograph* or Polygraph*).tw,kf. |
| 13 | or/10-12 |
| 14 | 4 and 9 and 13 |

### Emcare

| 1 | economic evaluation/ or exp health economics/ or "cost benefit analysis"/ or "cost control"/ or "cost effectiveness analysis"/ or "cost minimization analysis"/ or "cost of illness"/ or "cost utility analysis"/ |
| --- | --- |
| 2 | ((economic* adj1 (analysis or evaluat* or model*)) or (cost adj2 (effective** or utilit* or benefit or analysis or minimi*))).mp. or ("quality adjusted life year*" or qaly).tw,kw. |
| 3 | or/1-2 |
| 4 | exp sleep disordered breathing/ |
| 5 | Snoring/ |
| 6 | (apnoea or apnea or hypopnea or hypopnoea or snore or snoring).tw,kw. |
| 7 | ((sleep related or sleep-related) adj2 breathing disorder*).tw,kw. |
| 8 | ("upper airway resistance" or "sleep disordered breathing").tw,kw. |
| 9 | or/4-8 |
| 10 | exp polysomnography/ or diagnostic procedure/ |
| 11 | ((Limited-channel or limited channel or sleep or apnoea or apnea or portable or home or ambulatory) adj3 (study or test or device or diagnos* or monitor* or manage* or identif*)).tw,kw. |
| 12 | (Polysomnograph* or Polygraph*).tw,kw. |
| 13 | or/10-12 |
| 14 | 3 and 9 and 13 |

### PsychINFO

| 1 | exp Economics/ or exp Health Care Economics/ or exp "Costs and Cost Analysis"/ or *Evaluation/ or exp Health Care Costs/ |
| --- | --- |
| 2 | ((economic* adj1 (analysis or evaluat* or model*)) or (cost adj2 (effective** or utilit* or benefit or analysis or minimi*))).mp. or ("quality adjusted life year*" or qaly).ti,ab,id. |
| 3 | 1 or 2 |
| 4 | snoring/ or sleep apnea/ |
| 5 | (apnoea or apnea or hypopnea or hypopnoea or snore or snoring).ti,ab,id. |
| 6 | ("upper airway resistance" or "sleep disordered breathing").ti,ab,id. |
| 7 | ((sleep related or sleep-related) adj2 breathing disorder*).ti,ab,id. |
| 8 | or/4-7 |
| 9 | exp polysomnography/ |
| 10 | ((Limited-channel or limited channel or sleep or apnoea or apnea or portable or home or ambulatory) adj3 (study or test or device or diagnos* or monitor* or manage* or identif*)).ti,ab,id. |
| 11 | or/9-10 |
| 12 | 3 and 8 and 11 |

### ProQuest

| 1 | noft((("limited channel" OR "limited-channel" OR "sleep apnea" OR "sleep apnoea" OR portable OR home OR ambulatory) NEAR/2 (study OR test OR testing OR device OR diagnos* OR monitor* OR manage* OR identif*)) OR (polysomnography OR polygraph)) |
| --- | --- |
| 2 | AND (noft("upper airway resistance" OR "sleep disordered breathing" OR apnoea OR apnea OR hypopnea OR hypopnoea OR snore OR snoring) OR noft(("sleep related" OR "sleep-related") NEAR/2 ("breathing disorder*"))) |
| 3 | AND (noft(("quality adjusted life year*" OR qaly)) OR noft((cost NEAR/2 (effective* OR utilit* OR benefit OR analysis OR minimi*))) OR noft((economic* NEAR/1 (analysis OR evaluat* OR model*)) )) |
| 4 | #3 AND #2 AND #1 |

### CINAHL

| S1 | (MH "Costs and Cost Analysis+") OR (MH "Health Care Costs+") |
| --- | --- |
| S2 | (MH "Quality-Adjusted Life Years") OR (MH "Disability-Adjusted Life Years") |
| S3 | TI ( (economic* N1 (analysis or evaluat* or model*) ) OR ( ( cost N2 (effective** OR utilit* or benefit OR analysis OR minimi*) ) OR ( ("quality adjusted life year*" OR qaly) ) OR AB ( (economic* N1 (analysis OR evaluat* or model*) ) OR ( ( cost N2 (effective** or utilit* OR benefit OR analysis or minimi*) ) OR ( ("quality adjusted life year*" OR qaly) ) |
| S4 | S1 OR S2 OR S3 |
| S5 | TI ( ((sleep related or sleep-related) N2 breathing disorder*) ) OR TI ( "upper airway resistance" OR "sleep disordered breathing" ) OR TI ( apnoea OR apnea OR hypopnea OR hypopnoea OR snore OR snoring ) OR AB ( ((sleep related or sleep-related) N2 breathing disorder*) ) OR AB ( "upper airway resistance" OR "sleep disordered breathing" ) OR AB ( apnoea OR apnea OR hypopnea OR hypopnoea OR snore OR snoring ) |
| S6 | (MM "Sleep Apnea, Obstructive") OR (MH "Sleep Apnea Syndromes+") OR (MH "Sleep Apnea, Central+") OR (MH "Snoring") |
| S7 | S5 OR S6 |
| S8 | (MH "Polysomnography") |
| S9 | TI ( (("limited channel" OR "limited-channel" OR "sleep apnea" OR "sleep apnoea" OR portable OR home OR ambulatory) N2 (study OR test OR testing OR device OR diagnos* OR monitor* OR manage* OR identif*)) ) OR AB ( (("limited channel" OR "limited-channel" OR "sleep apnea" OR "sleep apnoea" OR portable OR home OR ambulatory) N2 (study OR test OR testing OR device OR diagnos* OR monitor* OR manage* OR identif*)) ) |
| S10 | S8 OR S9 |
| S11 | S4 AND S7 AND S10 |

### SCOPUS

| 1 | ( TITLE-ABS-KEY ( ( economic* W/1 ( analysis OR evaluat* OR model* ) ) OR ( cost W/2 ( effective* OR utilit* OR benefit OR analysis OR minimi* ) ) OR ( "quality adjusted life year*" OR qaly ) ) |
| --- | --- |
| 2 | AND TITLE-ABS-KEY ( ( "limited channel" OR "limited-channel" OR "sleep apnea" OR "sleep apnoea" OR portable OR home OR ambulatory ) W/2 ( study OR test OR testing OR device OR diagnos* OR monitor* OR manage* OR identif* ) ) |
| 3 | AND TITLE-ABS-KEY ( ( "upper airway resistance" OR "sleep disordered breathing" OR apnoea OR apnea OR hypopnea OR hypopnoea OR snore OR snoring ) OR ( ( "sleep related" OR "sleep-related" ) W/2 ( "breathing disorder*" ) ) ) ) |
| 4 | #3 AND #2 AND #1 |

### Cochrane Library

| 1 | (("limited channel" or "limited-channel" or "sleep apnea" or "sleep apnoea" or portable or home or ambulatory) NEAR/2 (study or test or testing or device or diagnos* or monitor* or manage* or identif*)):ti,ab,kw |
| --- | --- |
| 2 | ((polysomnograph* OR polygraph*)):ti,ab,kw |
| 3 | #1 or #2 |
| 4 | ((economic* NEAR/1 (analysis or evaluat* or model*)) or (cost NEAR/2 (effective** or utilit* or benefit or analysis or minimi*)) or ("quality adjusted life year*" or qaly) or ("disability adjusted life year*" or daly)):ti,ab,kw |
| 5 | ("upper airway resistance" OR "sleep disordered breathing" OR apnoea OR apnea OR hypopnea OR hypopnoea OR snore OR snoring) |
| 6 | (("sleep related" OR "sleep-related") NEAR/2 ("breathing disorder*")) |
| 7 | #5 or #6 |
| 8 | #3 and #4 and #7 |
